# Magnetic Bimerons in Cylindrical Nanotubes

**DOI:** 10.3390/nano13212841

**Published:** 2023-10-26

**Authors:** David Galvez, Mario Castro, Guilherme Bittencourt, Vagson Carvalho, Sebastian Allende

**Affiliations:** 1 Departamento de Física, CEDENNA, Universidad de Santiago de Chile, Santiago 9170124, Chile; 2 Departamento de Física, Universidade Federal de Viçosa, Viçosa 36570-900, Brazil; guilherme.bittencourt@ufv.br (G.B.);

**Keywords:** magnetic bimeron, phase diagram, micromagnetic simulation

## Abstract

This work presents the analysis of the stability of magnetic bimerons in a cylindrical nanotube. Through micromagnetic simulations, we study the influence of magnetic and geometrical parameters on the bimeron existence and size. The obtained results allow us to present diagram states showing the stability region of a bimeron as a function of the nanotube’s height and radius for different anisotropy and Dzyaloshinskii–Moriya interaction strengths. We also obtain two other magnetic states in the range of parameters where the bimeron is not stable: helicoidal and saturated states.

## 1. Introduction

Over the last two decades, magnetic topological textures have garnered significant attention from researchers, driven by their potential for technological application due to the inherent stability given by the topological protection that they offer [1]. One of the most important examples is the study of skyrmion, which consists of a magnetic texture characterized by an integer topological charge, *Q* [2]. The skyrmion with smaller energy has Q=1 and lives in magnetic bodies where the magnetization is perpendicular to the surface of the systems. Due to the appeal of using skyrmions in technological applications [3,4,5,6], their nucleation, stability, and dynamics have been studied in several contexts [7,8,9,10,11,12,13]. Besides skyrmions, other magnetic patterns present topological stability in both 2D [14,15,16,17] and 3D [18,19,20,21] systems. In particular, a particle-like topological structure that has recently been called to attention is the bimeron [14], also called in-plane skyrmion [22], which consists of a magnetization profile having two centers, in which a meron and an antimeron with opposite polarizations are positioned. The bimeron with the minimum energy consists of a structure with a total topological charge Q=1 as its skyrmion counterpart. Therefore, it belongs to a nontrivial topological sector, and consequently, it cannot be deformed continuously into a ferromagnetic state. Although skyrmions and bimerons have equal topological charges and are stabilized by the Dzyaloshinskii–Moriya (DM) interaction, they present some differences to be highlighted. For instance, unlike the skyrmions, bimerons are not cylindrically symmetric [22,23], which may have significant consequences in phenomena regarding static and dynamical properties of these magnetization collective modes. For instance, due to different deformations occurring during the displacement of skyrmions and bimerons along a nanotrack, bimerons can move longer distances than skyrmions before being annihilated in the track border due to the Magnus effect [24].

Recent works have analyzed the influence of curvature-induced effective interactions on the magnetic properties of skyrmions [25,26,27]. It was shown that curvature-induced magnetochiral effects make these topological spin textures increase/decrease their stability when they are hosted on curved manifolds [28,29,30,31]. Additionally, the geometry of a magnetic nanofilm can also influence the skyrmion shape [32] and their dynamics, which can be affected by curvature-induced forces [33,34,35]. Finally, it was also shown that, depending on the geometrical parameters of systems with azimuthal symmetry, skyrmions could be stabilized on cylindrical [36] or truncated conical [37] nanotubes. On the other hand, bimerons have been considered to be stabilized and guided only in planar geometries [14,22,38,39,40]. In these systems, the incorporation of interfacial DMI has demonstrated the feasibility of stabilizing skyrmions and transitioning them into bimerons (or vice versa) through the manipulation of the external magnetic field and perpendicular anisotropy [41,42]. Additionally, in helimagnets such as $Fe_{0.5}Co_{0.5}Ge$, the spontaneous formation of magnetic bimerons has been observed, which can be controlled by modifying the sample thickness [43]. The facts stated above, coupled with the distinctive static [22] and dynamical [24] properties of skyrmions and bimerons when hosted in planar systems, lead to an open question regarding the analysis of bimeron stability in curved magnetic structures.

In this work, we study the stability of bimerons in magnetic curved nanostructures. Specifically, we study bimerons in magnetic nanotubes as a function of geometrical and magnetic parameters. Because a cylindrical nanotube has no borders, it is an interesting curved system to nucleate and transport bimerons due to the possibility of avoiding their annihilation at the system’s border caused by the bimeron Hall effect (similar to the skyrmion Hall effect).

This article is organized as follows: In Section 2, we describe the method we adopted in the present work, and in Section 3, we present our results and discussions. Finally, in Section 4, we present our conclusions.

## 2. Models and Methods

To study the bimeron’s stability on nanotubes, we performed micromagnetic simulation using the mumax3 GPU accelerated code [44]. This code allows for the numerical solving of the Landau–Lifshitz–Gilbert (LLG) equation, written as

(1) $$\begin{array}{c} \frac{dm}{dt}=\frac{\gamma }{M_{s}}m\times \frac{\delta E}{\delta m}+\alpha m\times \frac{\partial m}{\partial t}, \end{array}$$

where $m=M/M_{s}$ is the normalized magnetization, $M_{s}$ is the saturation magnetization, γ is the gyromagnetic ratio, α is the damping factor, and $E=E_{ex}+E_{DMI}+E_{K}+E_{M}$ is the magnetic energy density, that includes the contributions coming from the exchange ($E_{ex}$), bulk Dzyaloshinskii–Moriya (DMI) ($E_{DMI}$), anisotropy ($E_{K}$), and self-magnetostatic ($E_{M}$) interactions.

**Figure 1:**
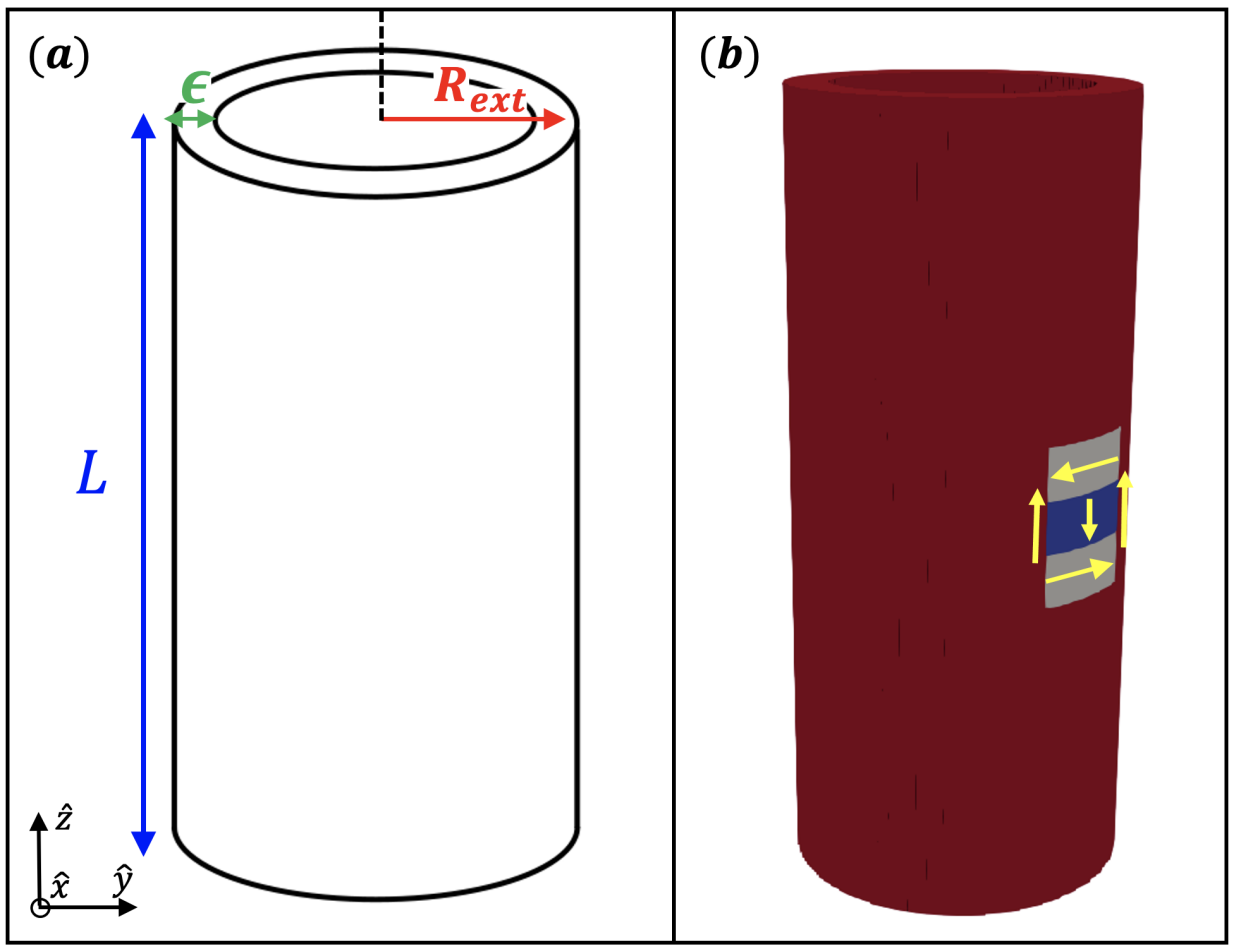
(**a**) Schematic representation of the considered structure and the adopted coordinate system. (**b**) Schematic view of the initial condition for the magnetization.

Initially, we perform micromagnetic simulations to obtain a stable bimeron on a nanotube described by the parameters of FeGe [45]. We consider a nanotube with an inner radius $R_{int}=24$ nm, outer radius $R_{ext}$ = 32 nm, and height *L* = 150 nm (see Figure 1a). When these parameters are modified, we will explicitly indicate their values. The magnetic parameters of FeGe are saturation magnetization $M_{s}=3.84\times 10^{5}$ A/m, exchange stiffness $A_{ex}=8.78$ p J/m, and a bulk DM constant D=2.0 mJ/m${}^{2}$. We also consider a uniaxial anisotropy with an easy axis along the $\hat{z}$ direction. These parameters determine an exchange length of $L_{ex}=\sqrt{2A_{ex}/\mu_{0}M_{s}^{2}}\approx 9.7$ nm. The discretization was 0.5×0.5×0.5 nm${}^{3}$, smaller than the exchange length.

In all simulations, in order to standardize the simulation process and ensure sufficient space for bimeron stabilization, we have consistently positioned the initial state in the center of the nanotube. The initial state was considered as follows: the magnetization has a direction $\hat{z}$ with an exception for a small region of the nanotube of size (20 nm, 20 nm, 30 nm) centered at $\left(R_{ext},R_{ext},0\right)/\sqrt{2}$. This small box was magnetized at $-\hat{z}$ in the center. The upper and lower boundaries of the box were magnetized along $-\hat{y}$ and $\hat{y}$, respectively. To pin the center of the bimeron during the relaxation process, we use anisotropy engineering effects [46] by defining a tiny cylindrical region in the center of the box. The tiny cylindrical region has a radius of 2.5 nm and high equal to 8 nm. In this tiny cylindrical region, we tilt the anisotropy in the radial direction with an angle of 15 degrees. We study the bimeron stabilization by changing the geometrical parameters of the nanotube, the anisotropy constant, and the bulk DM constant under an extensive range of parameters.

## 3. Results and Discussion

**Figure 2:**
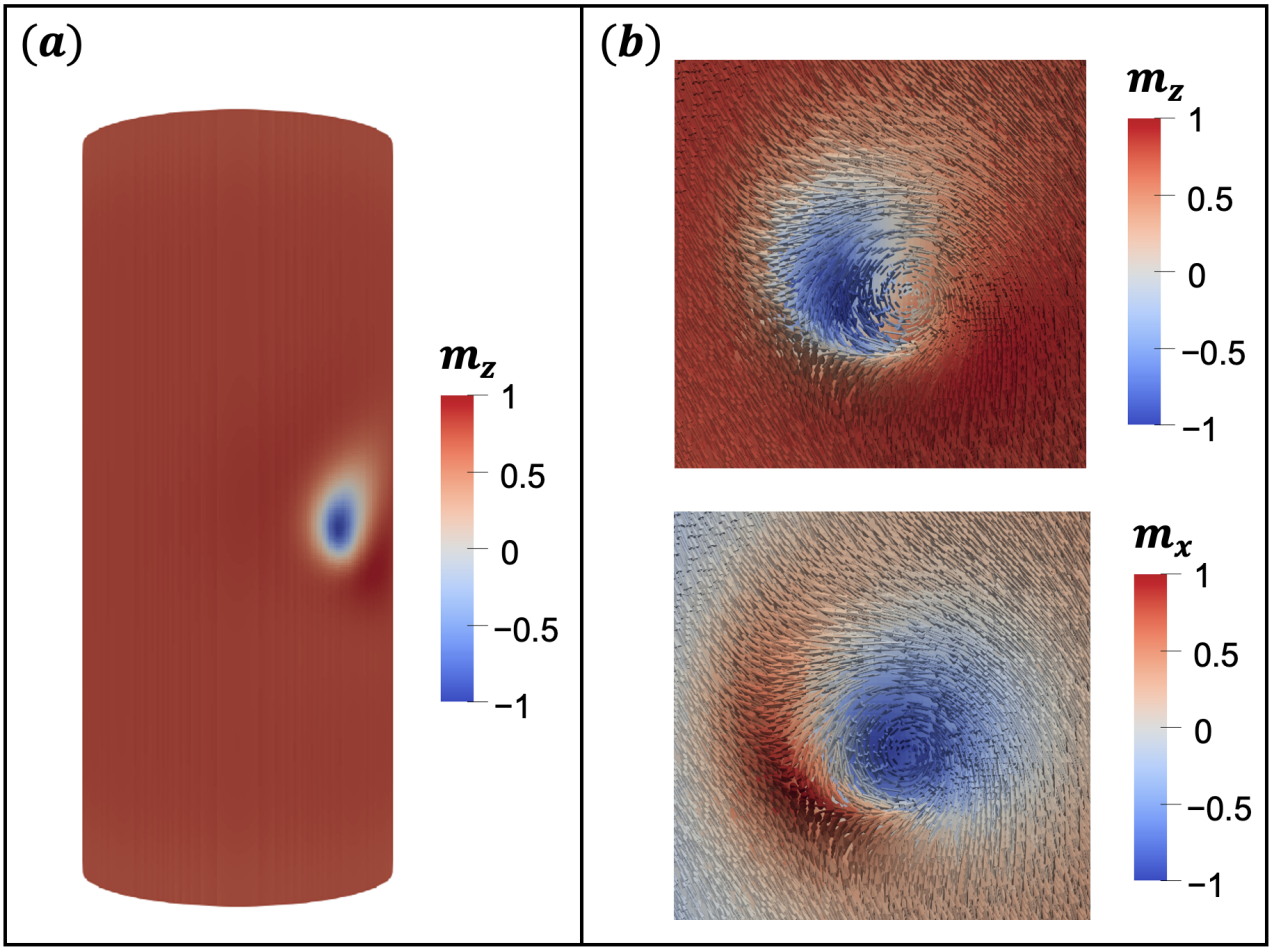
(**a**,**b**) Bimeron stabilized in a nanotube considering D=2.0 mJ/m${}^{2}$, K=170 J/m${}^{3}$ for a nanotube with L=150 nm, $R_{ext}=32$ nm, thickness ϵ=8 nm. The arrow indicates the magnetization direction.

Our simulations allow us to obtain a stable bimeron in a nanotube described by the parameters of FeGe, as shown in Figure 2. It can be noticed that the magnetization vector field in the inner region of the bimeron orientates in the opposite direction of the magnetization that is in the outer area, i.e., while magnetization points along the $\hat{z}$ direction out of the bimeron area, it points in the $-\hat{z}$ direction in the inner region, herein called the bimeron’s core.

After stabilizing a bimeron for specific magnetic and geometric parameters, we analyze the effects of varying *K*, *D*, and the geometric parameters on the bimeron stability.

### 3.1. Bimeron Stabilization as a Function of the Anisotropy Strength

**Figure 3:**
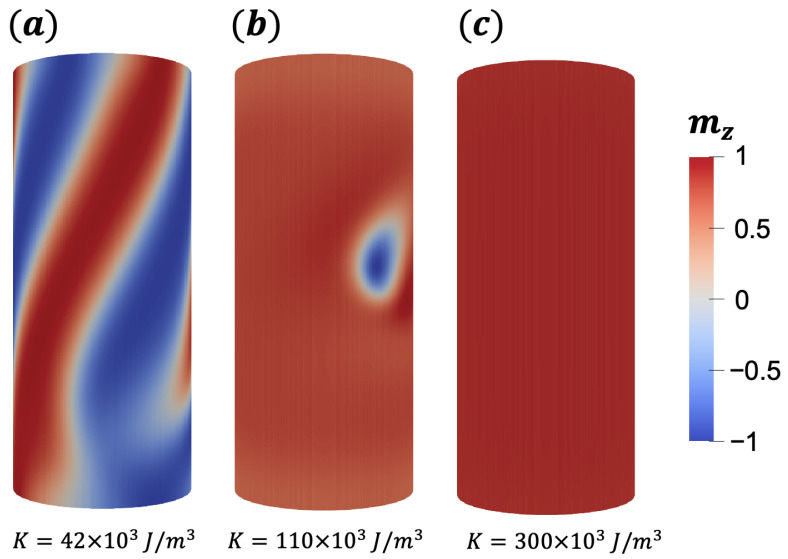
Final magnetic states as a function of the anisotropy value for a magnetic nanotube with L=150 nm, $R_{ext}=32$ nm, thickness ϵ=8 nm, and DM parameter D=2.0 mJ/m${}^{2}$; (**a**) shows the helicoidal state, while (**b**) depicts a bimeron; (**c**) shows the saturated state. The color bars refer to the $\hat{z}$ component of the magnetization.

After stabilizing a bimeron for a given set of magnetic parameters and a specific geometry (L=150 nm, $R_{ext}=32$ nm, and thickness ϵ=8 nm) and D=2.0 mJ/m${}^{2}$, we have analyzed the influence of the anisotropy strength on the bimeron properties. Therefore, we use the initial condition described in Section 2 and evaluate the final magnetization configuration after the system’s relaxation. In this case, the performed simulations consider a uniaxial anisotropy along the $\hat{z}$ direction, ranging from K=20 kJ/m${}^{3}$ to 400 kJ/m${}^{3}$. We can then evaluate the range of *K* stabilizing the bimeron and their properties as a function of the anisotropy strength. The results evidence that bimerons are stabilized for anisotropy values in the interval between K=60 kJ/m${}^{3}$ and K=250 kJ/m${}^{3}$. Figure 3 depicts the z component of the magnetization for different relaxed states and different values of *K*. Below the lower limit of the anisotropy where the bimeron is stable, there is the emergence of the helicoidal state (see Figure 3a) along all the nanotube’s surface. In this work, we will classify all the magnetic structures in which neither a bimeron nor a saturated state is observed as helicoidal states. This classification is based on the fact that these structures share similar properties of the helical states observed in planar magnetic systems with DMI [47,48]. This state is characterized by regions where the magnetization continuously changes from $\hat{z}$ to $-\hat{z}$ direction. The appearance of the helicoidal state is a natural consequence of the competition between the exchange and the strong intrinsic DM interaction, which is chiral, and the small uniaxial anisotropy. Similar states can also be observed in planar systems at low fields, where the ground state arises due to the competition between the DM and exchange interaction [47].

Above the upper anisotropy value, ensuring the stabilization of a bimeron, we observe a saturated state (see Figure 3c), which emerges due to the high anisotropy value that promotes the alignment of the magnetic moments along the longitudinal direction of the nanotube. Analog states can be observed for skyrmions stabilized in a nanowire structure [49].

**Figure 4:**
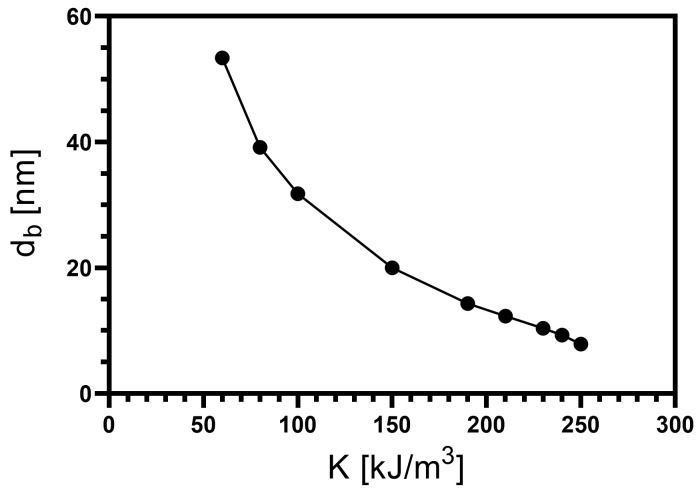
Bimeron’s core diameter ($d_{b}$) as a function of the anisotropy constant (*K*) for a magnetic nanotube with L=150 nm, $R_{ext}=32$ nm, thickness ϵ=8 nm, and DM parameter D=2.0 mJ/m${}^{2}$.

In the interval of anisotropy values for which the bimeron is stabilized, one can notice a change in the shape and size of the bimeron as a function of *K*. The smaller the *K*, the bigger the region the bimeron covers. Accordingly, an increase in *K* reduces the bimeron size until it disappears for K≈260 kJ/m${}^{3}$. Based on the above-described, we have analyzed the diameter of the bimeron’s core as a function of the anisotropy constant. We performed simulations varying the values of *K* inside the defined interval where the bimerons are stable, and for each case, we calculate the diameter of the bimeron’s core. Furthermore, we have considered that the cells belonging to the core are those whose normalized magnetization is $m_{z}\leq 0$. From the number of cells forming the core, we determine the effective area of the bimeron corresponding to the sum of the area of the cells’ face. Finally, we use an approximation where the bimeron is in a circle of area $A=\pi d_{b}^{2}/4$ and determine the approximated value for the bimeron’s core effective diameter ($d_{b}$) as a function of *K*, whose values are presented in Figure 4. As stated before, the bimeron’s core diminishes with *K*. It is worth noticing that, in the interval *K* = 60–190 kJ/m${}^{3}$, there is a faster decrease in the bimeron’s core than for *K* ranging from 190 kJ/m${}^{3}$ to 250 kJ/m${}^{3}$. Therefore, the increment of *K* forces the magnetic moments pointing along the nanotube axis, reducing the bimeron size.

**Figure 5:**
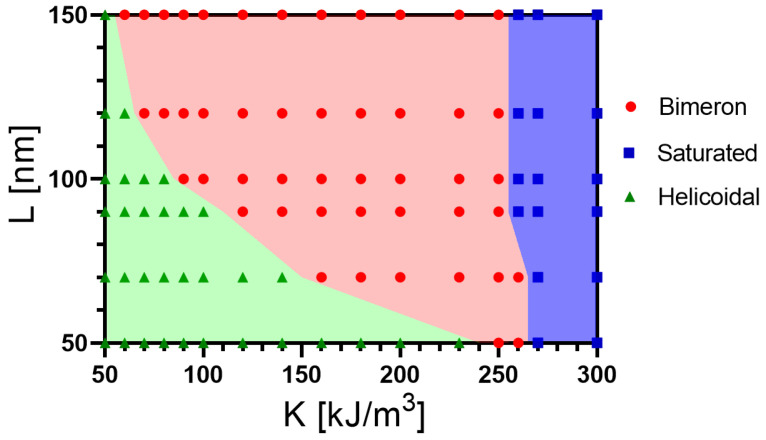
Magnetic state diagram L–K for a nanotube with $R_{ext}=32$ nm, ϵ=8 nm, and D=2.0 mJ/m${}^{2}$: the helicoidal (green triangles), the saturated (blue squares), and the bimeron states (red circles).

**Figure 6:**
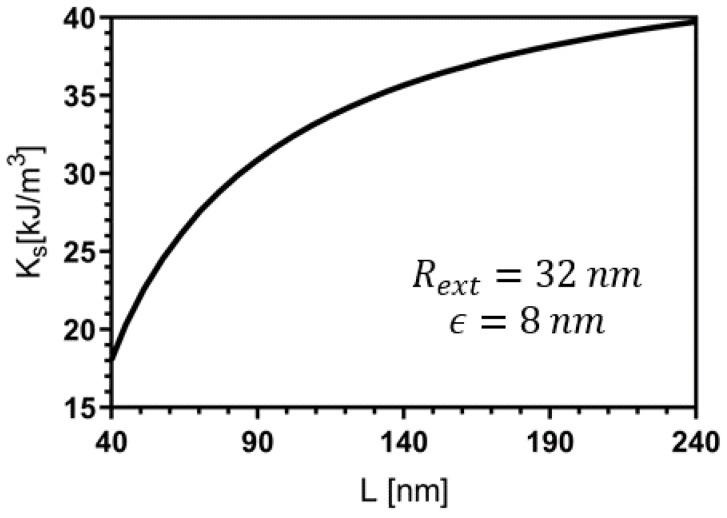
Shape anisotropy constant $K_{s}$ as a function of the length of the nanotube of FeGe when it is magnetically saturated. The external radius and thickness are 32 nm and 8 nm, respectively.

The competition between the uniaxial anisotropy, DMI, exchange, and shape anisotropy is fundamental for determining the bimeron stability and size. Therefore, we now analyze how the nanotube’s geometry affects the relaxed magnetic state. Firstly, we study the influence of the nanotube height (*L*) on the bimeron’s stability. In this case, the nanotube geometry determines the shape anisotropy, since a nanotube with a larger height has a strong shape anisotropy in the longitudinal direction. An increase in *L* can compensate for reductions in the uniaxial anisotropy, allowing the bimeron stabilization. In this context, we determine the region where one stabilizes a bimeron as a function on *L* and *K*. Figure 5 presents the obtained state diagram considering a nanotube with $R_{ext}=32$ nm, ϵ=8 nm, and D=2.0 mJ/m${}^{2}$, and *L* varying from 50 to 150 nm. In the state diagram, one identifies the magnetic states found previously in Figure 3, in that we observe one of the helicoidal (green triangles), the bimeron (red circles), and the saturated state (blue squares). Figure 5 evidences the existence of a minimum value of the anisotropy constant separating the bimeron from helicoidal configurations. This lower threshold depends on the nanotube length. Indeed, due to the increase in the shape anisotropy ($K_{s}$) as a function of *L*, we obtain that the lower threshold of *K* diminishes with *L*, as shown in Figure 5. In addition, for K≳250 kJ/m${}^{3}$, the values of the shape anisotropy force the magnetic moments to point in the nanotube axis, and the saturated state is stabilized. The behavior of $K_{s}$ as a function of *L* is presented in Figure 6, which evidences high variations in the value of $K_{s}$ for nanotubes with small values of *L* (between 45 and 100 nm).

We highlight that the upper thresholds of *K* that stabilize the bimeron are practically independent of the nanotube height. This aspect of the diagram state can be explained by the fact that the upper values of *K* are proportionally larger than $K_{s}$. Thus, this last contribution is negligible for the effective anisotropy along the nanotube longitudinal direction.

### 3.2. Bimeron Stabilization as a Function of the Dzyaloshinskii–Moriya Constant

**Figure 7:**
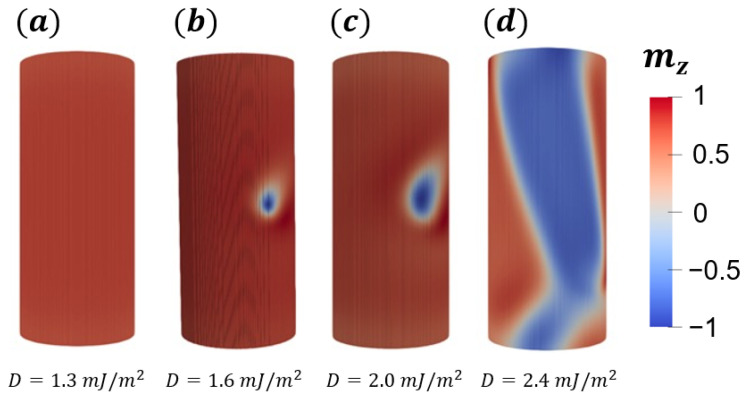
Final magnetic states as a function of the DM constant for a magnetic nanotube with L=150 nm, $R_{ext}=32$ nm, thickness ϵ=8 nm, and K=110 kJ/m${}^{3}$: (**a**) depicts a saturated state obtained for small values of *D*, while (**b**,**c**) present the bimeron state with a diameter dependent on *D*; (**d**) shows the helicoidal state obtained for large values of *D*. The color bars refer to the $\hat{z}$ component of the magnetization.

It is also interesting to analyze how changes in the DM constant influence the bimeron stability and size. Therefore, we have performed micromagnetic simulations considering a constant anisotropy (K=110 kJ/m${}^{3}$) and changing the DM constant, obtaining the range of *D* where the magnetic bimeron exists. Initially, we consider a nanotube with L=150 nm, $R_{ext}=32$ nm, and ϵ=8 nm. We have considered that *D* ranges from 1.3 mJ/m${}^{2}$ to 3.0 mJ/m${}^{2}$. Figure 7 shows the obtained relaxed states with their respective DM constant values. Our results show that bimerons can be stabilized for *D* ranging from 1.3 mJ/m${}^{2}$ to 2.3 mJ/m${}^{2}$. If the DM constant is below the minimum value of the stabilization range, we find the saturated state (Figure 7a). On the other hand, for D>2.3 mJ/m${}^{2}$, one notices the appearance of the helicoidal state, as presented in Figure 7d. Therefore, the states obtained are the same when we fixed the DM constant and varied the uniaxial anisotropy value. This behavior is also explained by the competition between anisotropy and DM interactions, which defines the stable state. That is, for small values of *D*, the uniaxial anisotropy is the main interaction of the system, and the lower energy state is the one minimizing this term to the total energy. On the other hand, the increase in *D* promotes the importance of the DM interaction to the stable state, and then, for high values of *D*, the helicoidal state takes place.

**Figure 8:**
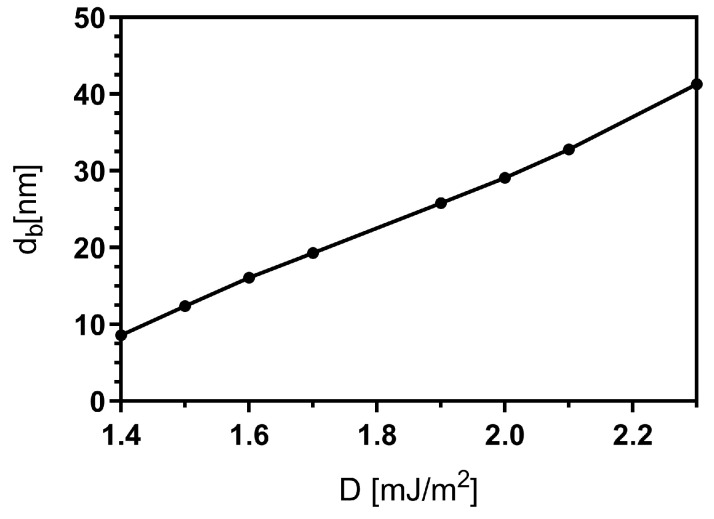
Bimeron’s core diameter ($d_{b}$) as a function of the DM constant (*D*) for a magnetic nanotube with L=150 nm, $R_{ext}=32$ nm, thickness ϵ=8 nm, and length L=150 nm.

The analysis of Figure 7b,c also reveals that changes in the DM constant affect the bimeron’s size and shape. Indeed, due to the intrinsic chirality originating from the DM interaction, the bimeron diameter increases with *D*. Therefore, we have studied the bimeron’s core diameter (following the procedure mentioned in Section 3.1) as a function of *D*. The main results are presented in Figure 8. The bimeron’s core diameter increases when we increase *D* because the DM interaction becomes the dominant term to the total energy when it is compared with the uniaxial anisotropy. Analogous behavior can be observed in the case of skyrmion in planar systems [50,51].

**Figure 9:**
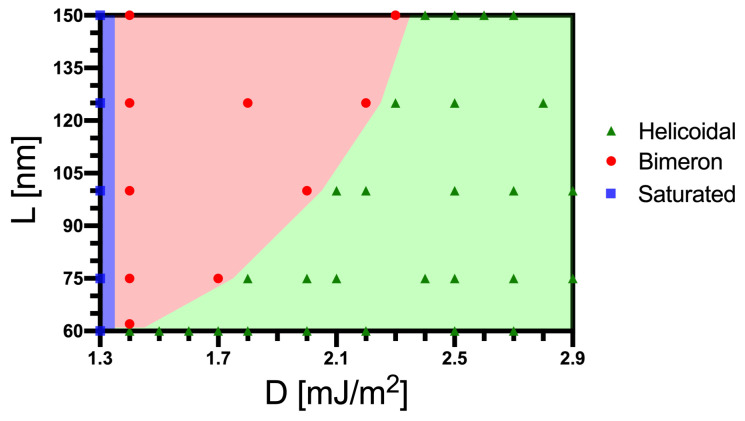
Magnetic state diagram L–D for a nanotube with $R_{ext}=32$ nm, ϵ=8 nm, and K=110 kJ/m${}^{3}$: the helicoidal (green triangles), the saturated (blue squares), and the bimeron states (red circles).

Aiming to show *L* influence on the bimeron stability for a specific DM parameter, we performed a series of simulations to obtain a state diagram presenting the final magnetic state of nanotubes with different lengths as a function of *D*. In this case, the external radius is 32 nm, thickness 8 nm, and the uniaxial anisotropy is fixed in 110 kJ/m${}^{3}$. Figure 9 presents the obtained relaxed states. Again, one can notice the emergence of the same three relaxed magnetic states obtained when we analyzed the system’s behavior as a function of anisotropy. Although the shape anisotropy does not affect the separation between the saturated and bimeron states for a specific *K*, it significantly affects the region between the bimeron and the helicoidal state. The increase in the shape anisotropy yields a larger region where the bimeron stabilizes.

**Figure 10:**
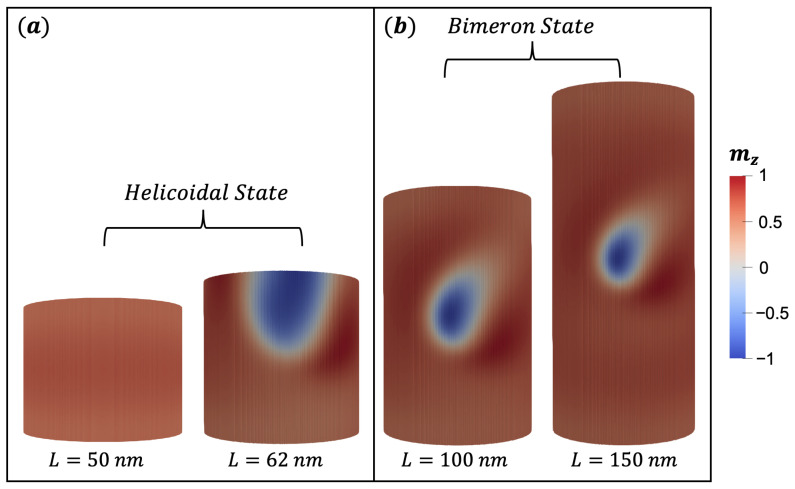
Magnetic configurations of FeGe for nanotubes with different heights: (**a**) shows helicoidal states, where left and right nanotubes illustrate a state with $m_{z}=0.89$ and an incomplete bimeron, respectively; (**b**) shows a complete bimeron with different core sizes for each nanotube. The color scale represents the magnetization component along the $\hat{z}$ direction.

It is important to mention that our article focuses on stabilizing bimerons, and so, any magnetic texture with a non-integer topological charge that is not saturated will be called herein a helicoidal state. For example, Figure 10 shows magnetic states for magnetic nanotubes with a fixed DM parameter D=2.0 mJ/m${}^{2}$, $R_{ext}=32$ nm, ϵ=8 nm, anisotropy constant K=110 kJ/m${}^{3}$, and the length is variated in 50, 62, 100, and 150 nm. Figure 10a shows that for L=50 nm, the magnetization of the nanotube is not saturated, i.e., $m_{z}=0.89$, which in the literature is part of a nanotube with helical configuration [52,53]. If we increase the length L=62 nm, we observe a magnetic state that does not present an integer bimeron, i.e., it presents an intermediate bimeron. Therefore, it would also be a part of the family of helicoidal states. Finally, by increasing the length by L=100 nm or L=150 nm, we observe the bimeron stabilization.

### 3.3. Bimeron Stabilization as a Function of the Geometric Parameters of the Nanotube

After analyzing the influence of magnetic parameters on the bimeron stabilization in magnetic nanotubes, we will study how the geometric parameters affect the bimeron properties. Firstly, we have analyzed the effects of the height in the bimeron stability in a nanotube with a fixed external radius $R_{ext}=32$ nm, fixed thickness ϵ=8 nm, fixed anisotropy constant K=110 kJ/m${}^{3}$, and fixed DM constant D=2.0 mJ/m${}^{2}$.

**Figure 11:**
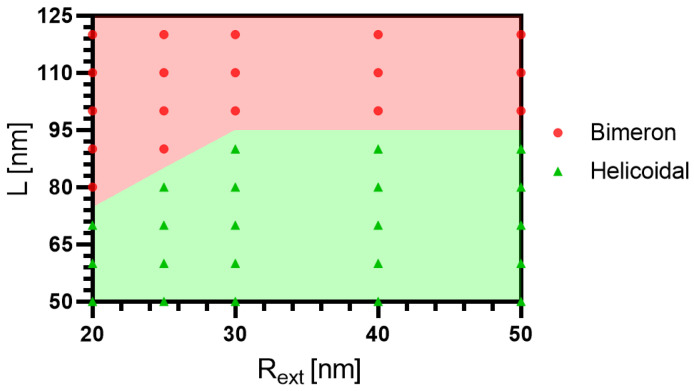
Magnetic state diagram L–R for a nanotube with ϵ=8 nm, K=110 kJ/m${}^{3}$, and D=2.0 mJ/m${}^{2}$: the helicoidal (green triangles), and the bimeron states (red circles).

Although the nanotube height influences the relaxed magnetization configuration, the external radius should produce essential changes regarding the range of parameters allowing the bimeron stabilization. Indeed, the nanotube presents a null Gaussian curvature, but its mean curvature depends on $R_{ext}$. Therefore, because the curvature induces effective exchange-driven anisotropy and DMI interactions [25,26], variations in $r_{e}$ can yield changes in the range of parameters stabilizing the bimeron. To corroborate our statements, we have performed micromagnetic simulations to analyze the influence of the external radius in the bimeron stabilization for nanotubes of FeGe with different heights. The considered range of external radius is between 20 nm and 50 nm, while *L* ranges from 50 nm to 120 nm. The results are presented in Figure 11, where one can notice that the bimeron is more stable for nanotubes with small $R_{ext}$. This fact can be explained by the emergence of an effective DMI induced by curvature [25,26], which favors the nucleation of the bimeron state. In this context, one can observe that the bimeron region increases in the interval $20≲R_{ext}≲30$ nm. For $R_{ext}≳30$ nm, we observe that the region where the bimeron is stable is almost not affected by the $R_{ext}$, because $d_{b}$ (core radius) is much smaller than the nanotube dimensions and then, locally, the bimeron lies on a quasi-planar surface.

## 4. Conclusions

Through micromagnetic simulations, we studied the stability of magnetic bimerons in cylindrical nanotubes for a range of magnetic and geometrical parameters. The results showed three magnetic relaxed final states: helicoidal, saturated, and bimeron. It was shown that the competition among uniaxial anisotropy, shape anisotropy, and DM interactions determines both the bimeron stability and size. We observe that an increase in the uniaxial anisotropy yields a decrease in the bimeron’s diameter. However, the DMI promotes the increase in the periodicity of the chiral structures [54], yielding the increase in the bimeron size, and, for considerable DMI strengths, the helicoidal state emerges. Additionally, the extra anisotropy along the longitudinal direction generated by the shape anisotropy increases the range of parameters where the saturated state is stable. Finally, we analyzed how the geometric parameters affect the bimeron stability. We have observed that the shape anisotropy originating from the increase in the nanotube height diminishes the bimeron stability as a function of the height. Nevertheless, there is a threshold value of *L* for which the bimeron stability is not more affected by the nanotube’s height. Additionally, due to the extra contribution given by the curvature-induced DMI, the bimeron is more stable in nanotubes with a small external radius. On the other hand, as $R_{ext}$ increases, the mean curvature diminishes so that for large values of $R_{ext}$, the bimeron stability is independent of the nanotube radius.

## Data Availability

All data and the simulation parameters that support this study are included within the article.

## Abbreviations

The following abbreviations are used in this manuscript:

DM	Dzyaloshinskii–Moriya
DMI	Dzyaloshinskii–Moriya interaction
LLG	Landau–Lifshitz–Gilbert
$d_{b}$	Bimeron’s core diameter
